# Design of a specific peptide against phenolic glycolipid-1 from *Mycobacterium leprae* and its implications in leprosy bacilli entry

**DOI:** 10.1590/0074-02760220025

**Published:** 2022-07-18

**Authors:** Nelson Enrique Arenas, Gilles Pieffet, Cristian Rocha-Roa, Martha Inírida Guerrero

**Affiliations:** 1 Hospital Universitario, Centro Dermatológico Federico Lleras Acosta, Bogotá, Colombia; 2 Universidad de los Andes, Departamento de Química, Bogotá, Colombia; 3 Universidad del Quindío, Facultad de Ciencias de la Salud, Grupo de Estudio en Parasitología y Micología Molecular-GEPAMOL, Armenia, Quindío, Colombia

**Keywords:** leprosy, Mycobacterium leprae, PGL-1, antimicrobial peptide, drug design

## Abstract

**BACKGROUND:**

*Mycobacterium leprae*, the causative agent of Hansen’s disease, causes neural damage through the specific interaction between the external phenolic glycolipid-1 (PGL-1) and laminin subunit alpha-2 (LAMA2) from Schwann cells.

**OBJECTIVE:**

To design a LAMA2-based peptide that targets PGL-1 from *M. leprae*.

**METHODS:**

We retrieved the protein sequence of human LAMA2 and designed a specific peptide using the Antimicrobial Peptide Database and physicochemical parameters for antimycobacterial peptide-lipid interactions. We used the AlphaFold2 server to predict its three-dimensional structure, AUTODOCK-VINA for docking, and GROMACS programs for molecular dynamics simulations.

**FINDINGS:**

We analysed 52 candidate peptides from LAMA2, and subsequent screening resulted in a single 60-mer peptide. The mapped peptide comprises four β-sheets and a random coiled region. This peptide exhibits a 45% hydrophobic ratio, in which one-third covers the same surface. Molecular dynamics simulations show that our predicted peptide is stable in aqueous solution and remains stable upon interaction with PGL-1 binding. In addition, we found that PGL-1 has a preference for one of the two faces of the predicted peptide, which could act as the preferential binding site of PGL-1.

**MAIN CONCLUSIONS:**

Our LAMA2-based peptide targeting PGL-1 might have the potential to specifically block this key molecule, suggesting that the preferential region of the peptide is involved in the initial contact during the attachment of leprosy bacilli to Schwann cells.

Hansen’s disease (leprosy) is an ancient infection that remains a significant health impairment in susceptible populations and is still endemic in several countries, such as Brazil, India, and Colombia.^1^ The long-term vision of the World Health Organization is to eradicate leprosy by 2030. The strategy of controlling leprosy still must be reinforced with new diagnostic tools in combination with improved therapeutic regimens.^2^ Moreover, the risk of drug resistance remains a latent threat; thus, cautious surveillance is necessary for preventing the spread of drug-resistant strains.^3^ Innovative therapies have been proposed as strategies to combat infection and antibiotic resistance by targeting pivotal bacterial processes, such as adhesion, cell wall permeability, quorum sensing, virulence regulons, and toxin production.^4^ In mycobacteria, this approach has been explored only in *Mycobacterium tuberculosis* and *Mycobacterium marinum*, and the approach targeted the PhoPR regulon, SapM, and ESX-1 secretion system.^4^ ^,^ ^5^ ^,^ ^6^ Some promising candidates are in the preclinical stages and are being tested in animal models.

Hansen’s disease is characterised by loss of sensitivity at the peripheral nerve level due to irreversible tissue damage and subsequent weakening by the infection chronicity.^7^ This process begins with the invasion of the causative agent, *Mycobacterium leprae*, through a specific interaction of phenolic glycolipid-1 (PGL-1) with human laminin subunit alpha-2 (LAMA2) to promote the attachment of mycobacterial to the basal lamina of Schwann cells and pathogen internalisation.^8^

The PGL-1 molecule is surface exposed in the mycobacterial cell wall and capsule, and its structure is composed of trisaccharide units, which are defined as methyl-rhamnose derivatives bound to a phenyl group, a mycocerosic acid, and a phthiocerol region.^9^ Since PGL-1 is a well-known diagnostic marker for Hansen’s disease, this molecule has been useful for the specific differentiation of *M. leprae* from other mycobacteria or even for the quantification of bacterial loads to monitor the treatment outcome during multibacillary infection.^10^ Furthermore, the PGL-1 molecule has been reported to induce a proinflammatory response and nerve damage in patients by inducing the activation of nitric oxide synthase in infected macrophages.^11^

PGL-1 binds specifically to the laminin multiprotein complex of the axon and is among the first steps during the Schwann cell interaction.^12^ LAMA2 is involved in Schwann cell differentiation and is a key component that mediates cell-surface interaction, migration, and assembly into tissues through the promotion of laminin connections with other extracellular matrix components.^13^ We hypothesised that dissecting the LAMA2 subunits into peptides could target the specific region that binds PGL-1 and provide a further application for therapeutic or diagnostic purposes. In this study, we addressed this strategy to design *in silico* a LAMA2-specific peptide that targets the PGL-1 molecule from *M. leprae*.

## MATERIALS AND METHODS

*Peptide design and parameters* - We retrieved the LAMA2 sequence from the UniProt database (accession code P24043) and evaluated peptide properties by using the antimicrobial peptide calculator implemented in the Antimicrobial Peptide Database (APD, https://aps.unmc.edu/home).^14^

We defined screening parameters based on peptides that were 60 residues long and included properties that were expected to support lipid binding, such as the following: hydrophobic ratio percentage, total net charge, GRAVY (grand average hydropathy value of the peptide), Wimley-White whole-residue hydrophobicity of the peptide, protein-binding potential (Boman index) and the total hydrophobic residues on the same surface. The 60-residue peptide was designed to preserve the functional regions of LAMA2, ensuring full PGL-1 coating within an exposed protein area; preferentially, the peptide was without disulfide bonds or any posttranslational modification and had a long peptide size to reduce the occurrence of alternative biological activity due to its length. We preferred to avoid bulky carbohydrate modifications since they might hinder contact with extracellular ligand molecules from the cell surface.

Since the expected PGL-1 molecule displayed a low solubility in aqueous solutions, we selected the peptide based on the best hydrophobic scores, and the key criteria was that peptides with positive values, compared to those with negative values, are more hydrophobic and thereby less soluble.^15^ Another parameter was the Wimley-White whole residue hydrophobicity; more negative values for peptides indicate a higher hydrophobicity.^16^ Other properties, such as the highest hydrophobic ratio percentage and the maximum number of hydrophobic residues on the same surface, were considered key for peptide selection.

LAMA2 was scanned for domain and functional motifs in SMART and visualised in the DOG program.^17^ ^,^ ^18^ The DISULFIND server (http://disulfind.disi.unitn.it/) was used to predict the disulfide bridges between cysteines and their connectivity pattern.^19^ A prediction for N-glycosylation was performed in NetNGlyc 1.0 (https://services.healthtech.dtu.dk/service.php?NetNGlyc-1.0) based on the consensus sequence Asn-Xaa-Ser/Thr.^20^

*Prediction of possible biological properties* - The peptide with the best score was checked for allergenicity using AlgPred 2.0 (https://webs.iiitd.edu.in/raghava/algpred2/index.html),^21^ toxicity in ToxinPred (https://webs.iiitd.edu.in/raghava/toxinpred/algo.php),^22^ and hemolytic activity by HemoPred (http://codes.bio/hemopred/).^23^

*Three-dimensional structure of the peptide* - The peptide was mapped in the LAMA2 protein and modeled by using I-TASSER^24^ and AlphaFold2 (https://colab.research.google.com/github/sokrypton/ColabFold/blob/main/AlphaFold2.ipynb).^25^ The AlphaFold2 method as implemented in Google ColabFold was used as suggested by Mirdita et al.,^26^ which differs from the original implementation from Deepmind^27^ by replacing the homology detection of AlphaFold2 with MMseqs2 (many-against-many sequence searching).^28^ This 3D model was subjected to a minimisation stage using the GROMACS package^29^ for 50000 steps using a steep descent algorithm, with a maximal force tolerance of 1000 kJ mol^-1^ nm^-1^. The peptide was optimised using the amber99sb-ildn force field^30^ and solvated in a dodecahedron box using the TIP3P water model.^31^ Na^+^ and Cl^-^ ions were added to neutralise the system’s charges and to reach a NaCl physiological concentration of 0.15 The stereochemical quality of the model before and after the minimisation stage was inspected using the Ramachandran plot, which was obtained using the Molprobity web tool (http://molprobity.biochem.duke.edu/).^32^

*Molecular dynamics simulation of PGL-1 binding to the peptide* - We carried out molecular dynamics simulations with the aim of inspecting the modes of interaction between the proposed peptide and the *M. leprae* trisaccharide PGL-1. The peptide-PGL-1 complex was obtained from molecular docking calculations using AUTODOCK VINA software;^33^ for this, the structure of PGL-1 was retrieved from the PubChem database (CID: 45480571). The search box was configured in such a way that it covered the entire surface of the peptide. The built complex with the best pose predicted by AUTODOCK VINA was subjected to MD simulations with the GROMACS 2019 package.^34^ The amber ff99sb-ILDN force field and the TIP3P model were used to represent the behavior of protein in water as a solvent. PGL-1 was parameterised using the ACPYPE web server (https://www.bio2byte.be/acpype) to obtain ligand parameters for GROMACS.^35^ The complex was neutralised with Na^+^ and Cl^-^ ions, brought to a concentration of 0.15 M NaCl and then subjected to a potential energy minimisation step for 50,000 steps (similar to that used for peptide minimisation), followed by two equilibration steps, including one NVT (constant volume and temperature) and a series of NPT (constant pressure and temperature) equilibrations, which were carried out for 250 ps using position restrictions on all heavy atoms. Finally, a production stage of 1000 ns (1 μs) was carried out, with a temperature of 310 K, which was controlled with the V-rescale thermostat, and a 1 bar pressure, which was controlled with the Parrinello-Rahman barostat. A time step of 2 fs was used. As a control, the peptide in water was also simulated following the same procedure. All visualisations were created with Chimera UCSF.^36^

*Prediction of dimer/PGL-1 interactions* - Using the minimised structure of the peptide, peptide association and oligomerisation were calculated by an *ab initio* strategy with the Galaxy-Homomer server (http://galaxy.seoklab.org/index.html).^37^ This server calculates the interface area (Å^2^) between predetermined chains (by user) Molprobity score^33^ and a docking score, in which high values determine a greater probability of peptide interaction and the model quality, respectively. As a preliminary method in which the predicted homodimer structure was used, we carried out molecular docking of PGL-1 following the same procedure used for the single peptide. Peptide oligomer interactions and homodimer/PGL-1 interactions were assessed with Ligplot+ software.^38^

## RESULTS

**Fig. 1:**
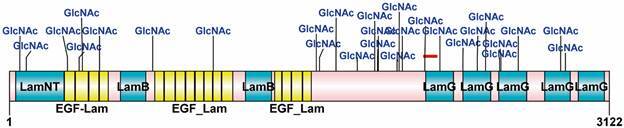
modular structure of laminin subunit alpha-2 from *Homo sapiens* (Human). N-linked glycosylation (GlcNAc) labeled asparagine (Asn) positions 55, 89, 303, 363, 380, 470, 746, 1061, 1597, 1614, 1700, 1810, 1901, 1916, 1920, 2017, 2028, 2045, 2126, 2240, 2360, 2435, 2478, 2551, 2558, 2648, 2868, and 2893 as predicted in NetNGlyc 1.0. The peptide position is shown in the red line above the first LamG domain.

*Structural features of the laminin subunit alpha 2* - To understand the structural features of the LAMA2 protein, domain mapping and functional motif analysis were performed along with analysis of the whole protein. Our prediction found 28 sites of N-glycosylation in LAMA2, which agrees with the functional annotation in the UniProt database. LAMA2 analysis in the SMART tool allowed us to identify the modular composition of four domains, including a single LamNT domain and the modular arrangement of EGF-Lam, laminin B (LamB), and laminin G (LamG) domains (Fig. 1). The LamNT domain is located between residues 33-285 through the N-terminus for protein insertion in the cell membrane. In contrast, LAMA2 contains 16 EGF-laminin domains, which are characterised by the presence of many cysteine residues that form disulfide bonds. We predicted 66 disulfide bonds that were distributed across the whole protein (data not shown). Furthermore, our analysis showed two LamB domains located between residues 578-710 and 1229-1364 that are interspaced by a set of EGF laminin domains. The C-termini exhibit an arrangement of five LamG domains at the C-terminus of the LAMA2 protein.

*Peptide selection and pharmacological properties* - LAMA2 contained 3,122 amino acids, and our computational screening resulted in 52 candidate peptides. However, based on our computer-aided peptide design strategy and physicochemical criteria, a 60-mer peptide was designed with a predominance of hydrophobic residues that are solvent-exposed to ensure a potential hydrophobic interaction with PGL-1 (Table). Thus, the calculated percentages of the most frequent amino acids were Val ratio = 13%, Lys = 10%, Gly = 10%, Leu = 8%, and 7% for Ile, Phe, Ala, Ser, and Asn residues, respectively. This designed peptide showed a hydrophobic ratio of 45%, in which 22 hydrophobic residues (37%) were located on the same surface. Other calculated parameters included 0.18 as a GRAVY value, a Wimley-White whole-residue hydrophobicity of the peptide of 7.94, and a protein-binding potential of 0.78 (Table). The peptide mapping indicated that our peptide was located between the 2161-2220 positions of the LAMA2 protein, specifically in the first LamG domain (positioned between 2166-2311), and seemed to be surface exposed in the three-dimensional structure.

Other pharmaceutical properties of our peptide indicated that it was nonallergenic (score: 0.31), nontoxic (-1.00), and nonhemolytic.

**TABLE:** List of predicted peptides from the human LAMA2 protein

Region	Hydrophobic ratio (%)	Total net charge	GRAVY	Wimley-White whole-residue hydrophobicity	Protein binding Potential (Kcal/mol)	Total hydrophobic residues on the same surface
1-60	43	+2.2	0.09	6.84	0.73	14
61-120	27	+3.5	-1.09	16.95	2.45	7
121-180	40	-2.5	-0.16	6.19	1.18	15
181-240	28	-3.5	-0.58	15.76	1.95	8
241-300	42	+4.5	-0.22	9.70	2.11	9
301-360	33	-3.2	-0.93	20.63	2.57	0
361-420	32	+4.2	-0.47	9.78	1.93	0
421-480	32	+2.5	-0.66	16.97	2.31	ND
481-540	35	-2.0	-0.60	10.36	2.01	7
541-600	33	-0.7	-0.51	7.97	1.44	2
601-660	35	-13.5	-0.37	24.8	1.90	13
661-720	45	+2.5	0.26	5.8	1.06	21
721-780	35	-3.25	0.40	14.49	1.87	ND
781-840	32	-2.7	-0.28	8.34	1.08	ND
841-900	32	-2.0	-0.34	10.81	1.57	2
901-960	37	+0.25	0.55	14.78	2.17	9
961-1020	37	-0.5	0.45	10.53	1.73	2
1021-1080	33	+2.2	-0.49	10.91	1.77	ND
1081-1140	33	+1.5	-0.63	12.98	2.23	2
1141-1200	38	+0.25	-0.21	12.42	1.48	ND
1201-1260	40	0	-0.35	10.71	1.00	9
1261-1320	32	+0.75	-0.66	17.98	2.27	7
1321-1380	37	-1.5	-0.32	15.36	2.04	13
1381-1440	32	-0.75	-0.29	8.23	1.40	4
1441-1500	38	-1.5	-0.22	9.06	1.55	ND
1501-1560	27	-3	-0.60	14.06	1.66	ND
1561-1620	43	-2.5	-0.15	9.86	0.92	18
1621-1680	32	-1.75	-0.75	24.7	2.62	14
1681-1740	37	-2	-0.91	33.88	3.02	17
1741-1800	37	-5	-1.04	36.57	3.1	18
1801-1860	35	-4	-0.83	30.02	2.81	16
1861-1920	35	-5.75	-0.70	27.67	2.41	17
1921-1980	40	+1.25	-0.55	23.59	1.92	20
1981-2040	35	+1.25	-0.85	25.72	2.69	17
2041-2100	38	+2.25	-0.64	23.44	2.2	18
2101-2160	32	+2	-0.85	27.87	2.65	14
2161-2220	45	+2	0.18	7.94	0.78	22
2221-2280	35	-0.5	-0.17	7.69	1.64	11
2281-2340	33	-1	-0.44	17.35	1.64	9
2341-2400	43	+1.25	0.07	3.61	1.4	15
2401-2460	30	-1.75	-0.62	12.57	2.16	12
2461-2520	32	+3	-0.45	13.76	1.74	6
2521-2580	32	+2	-0.23	13.95	1.49	7
2581-2640	35	+4	-0.42	15.12	2.42	12
2641-2700	40	0	-0.25	14.14	1.37	13
2701-2760	38	-4.5	-0.31	22.51	1.29	ND
2761-2820	42	+4.5	-0.21	13.71	2.02	15
2821-2880	30	+1.5	-0.58	11.66	1.77	11
2881-2940	40	+0.5	0.04	7.53	1.13	ND
2941-3000	42	-2.7	0.24	7.55	0.56	14
3001-3060	33	-1.2	-0.58	16.92	1.92	8
3061-3120	39	+3	-0.07	11.44	1.12	ND

ND: nondetermined.

**Fig. 2:**
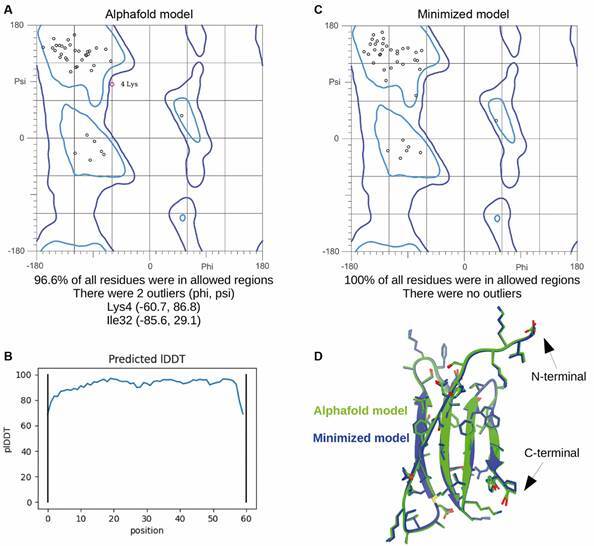
results for the Ramachandran plot for the structure of the selected peptide before (top-left side) and after energy minimisation (top-right side). On the bottom side, the superposition of the structures before (green) and after (blue) the minimisation are shown. The N- and C-termini are shown schematically. The peptide is shown as ribbons with all its residues as sticks.

*Peptide 3D structure* - The peptide modeling was based on artificial intelligence through Alphafold. The predicted structure exhibited a high structural quality according to its Ramachandra plot, which indicates that 96.6% of the residues have a favorable stereochemistry (Fig. 2A). In addition, AlphaFold provided two confidence indicators to determine the reliability of the results. The first indicator, called pLDDT (predicted lDDT-Cα), provided a measurement of the local confidence (for each residue) on a scale from 0 to 100. The results presented in Fig. 2B show that most of the residues had a plDDT > 80, which corresponded to confidence ranging between high and very high. The second indicator, called PAE (Predicted Aligned Error), represented the expected error associated with the relative positions of the different domains of the protein. The error values calculated for the peptide were consistently low except for the first two and last two residues (data not shown), thus indicating good confidence in the positions of the beta-strands. Even so, after a minimisation step, a structure with improved structural quality was obtained based on its Ramachandran plot, which indicates that all (100%) of the amino acids have favorable stereochemistry (Fig. 2C). An overlay of the peptide structure before and after minimisation is shown in Fig. 2D. In general, the peptide exhibited a random coiled region in the N-termini followed by four antiparallel β-strands. Our peptide lacked Cys residues, and its three-dimensional structure resembled a defensin-like beta structure without N-glycosylation sites. Additionally, the sequence of our peptide has 89.92% sequential identity, which corresponds to the crystal structure of the LG1-3 region of LAMA2 (PDB 1QU0) from *Mus musculus*.

**Fig. 3:**
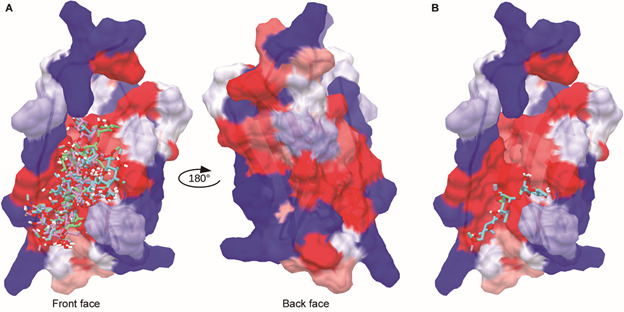
complete results of molecular docking triplicate (A-left) and the back side of the peptide (A-right). The first pose of each molecular docking run is shown in blue, pink and green (B). The protein is shown as a surface, and the residues are coloured according to their hydrophobicity, with blue being very hydrophilic and red being very hydrophobic. PGL-1 is shown in sticks.

*Docking and molecular dynamics analysis* - The peptide-ligand complex was obtained by molecular docking calculations. Given the stochastic nature of AutoDock Vina’s search algorithm, we performed a triplicate run. All 27 conformations (nine for each run) were located in the same hydrophobic region of the chosen peptide, as shown on the left side of Fig. 3A. The foregoing suggests that the PGL-1 ligand would have a greater affinity or a greater preference to interact with this face of the peptide (from now on, this face will be called the front face of the peptide) than with the back face, which has a more hydrophilic character (Fig. 3A-right). Fig. 3B shows the best pose obtained for each run, and the best pose had a binding affinity value of -5.1 kcal/mol ± 0.0. One of these conformations was used as the initial coordinates of the peptide/PGL-1 complex for the MD simulations.

**Fig. 4:**
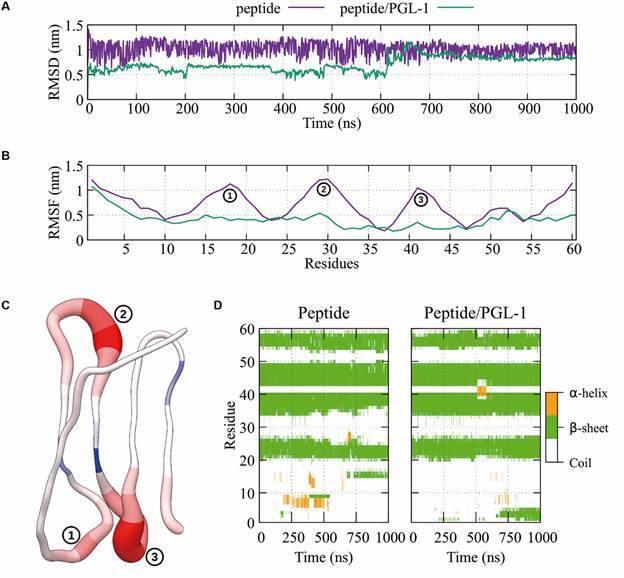
(A) RMSD of the backbone and (B) RMSF of the protein residues for the simulations with (green line) and without PGL-1 (purple line). (C) Peptide B-factor ratio. Blue and red represent the increase in movement for the residues in the peptide with and without PGL-1, respectively. Similarly, a thicker ribbon indicates greater flexibility. (D) Secondary structure as a function of time of the protein with and without PGL-1. α-helix, beta sheets and disordered regions are shown in orange, green and white, respectively.

In our MD simulations, we first inspected the structural stability of the peptide (Fig. 4). The RMSD results (Fig. 4A) suggest that the peptide without PGL-1 was more flexible during the simulation, or the peptide interaction with PGL-1 favors conformational changes. These greater changes in the RMSD of the peptide without PGL-1 are associated with greater flexibility in the N-termini coil region, as shown in Fig. 4B. The fluctuations (RMSF) of the loop regions in the peptide without PGL-1 were considerably greater than those in the peptide/PGL-1 complex (Fig. 4B-C). Additionally, we tracked the secondary structure of the peptide (Fig. 4D), suggesting that the main structure of four antiparallel beta sheets is highly stable since after 1000 ns of simulation, the beta sheets were preserved in the presence and absence of PGL-1. It was also observed that after approximately 650 ns, a new beta sheet was formed at the N-terminal end of the peptide by PGL-1 binding (Fig. 4D), which may be related to the fact that the RMSD of the peptide in complex with PGL-1 stabilises at ~0.8 nm.

To map the modes of interaction between PGL-1 and the selected peptide, we calculated the contact frequencies between them by defining each contact within a range of 3 Å in the interaction.

**Fig. 5:**
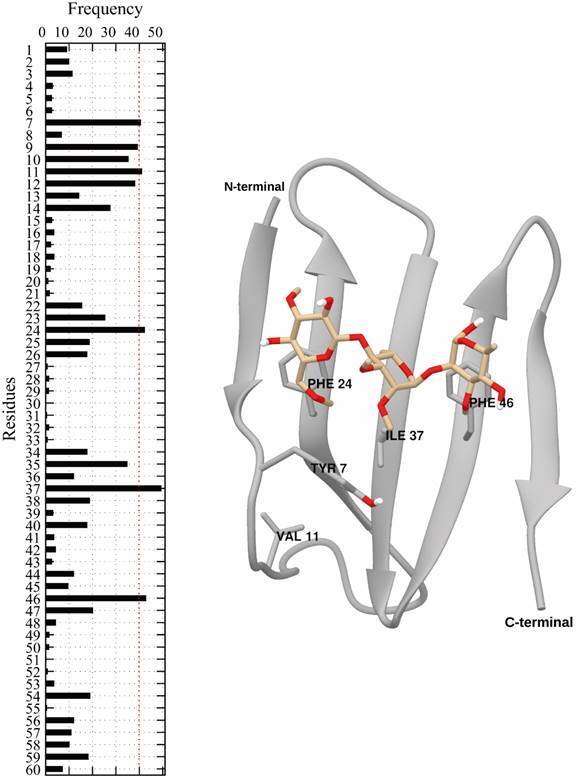
interaction frequency in the peptide/PGL-1 complex (left). Schematically, the last conformation of the simulation is shown, highlighting the main interactions between the peptide and PGL-1 (right). The N- and C-termini are shown schematically. The peptide is illustrated with ribbons, while the main residues and PGL-1 are represented by sticks.

As shown in Fig. 5, the highest interaction frequencies (from 40% to 50%), which define a hydrophobic pocket that is delineated by residues Tyr7, Val11, Phe24, Ile37 and Phe46. Tyr7 and Val11, are located in the loop of the N-terminal end, and the other three residues Phe24, Ile37 and Phe46 are part of the peptide front face, and each is located in a different beta sheet. Additionally, several residues, such as Leu22, Tyr25, Ala36, Glu38, Ser45 and Leu47, present interactions of less than 20%. Those residues are located on the rear face of the peptide in the preferential face of PGL-1 to interact with the peptide front face.

**Fig. 6:**
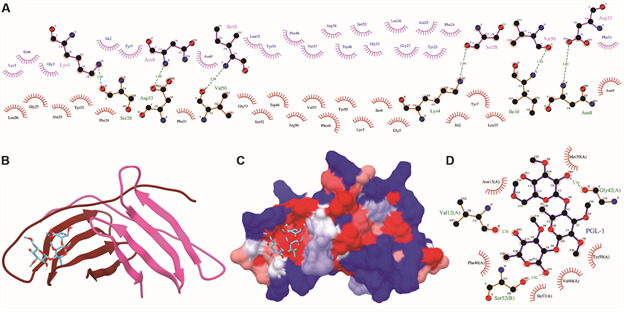
(A) 2D interactions between peptide chains in the homodimer. Structure of the complex formed by the homodimer and PGL-1 using ribbons (B) for the homodimer and hydrophobic surface (in which blue indicates the most hydrophilic residues, red indicates the most hydrophobic residues) (C); in both cases, sticks are used for PGL-1. (D) 2D interaction between the homodimer and PGL-1. (A and B) Chains A and B are shown in red and pink, respectively. (A and D) Hydrogen bonds are shown as green dashed lines, and hydrophobic interactions are shown as half circles with lines through them.

*Homodimer interactions of LAMA2-derived peptide with PGL-1* - Oligomer modeling results were used to calculate the potential peptide-peptide interactions. Oligomer calculations were based on the structure of a laminin G-like module of LAMA2 (peptide coverage between 2157-2216), leading to a homodimer of 7410.55 Å^2^ (Fig. 6C). Peptide interacting chains were analysed in Ligplot+ software to calculate dimer interactions. Interacting chains might be associated with 26 nonbonded contacts that involve neutral, aliphatic, aromatic, and positively charged residues (Fig. 6A). The molecular docking result for PGL-1 in the homodimer docks at a site with similar hydrophobicity to that of the peptide alone brought the mycobacterial ligand together (Fig. 6B-C). We inspected the interactions, which included hydrophobic interactions with residues such as Asn13, Ile37, Met39, Val44, Phe46, and Tyr59, all of which were in chain A, and hydrogen bond interactions with Val12 and Gly42 in the A chain and with Ser52 in chain B (Fig. 6D).

## DISCUSSION

Since ancient times, Hansen’s disease has been a public health problem worldwide. Despite the many efforts to eradicate this disease, concerns over concomitants, such as poverty, delayed diagnosis, and drug resistance, have emerged.^2^ ^,^ ^39^ ^,^ ^40^ The PGL-1 molecule has significantly contributed to the serodiagnosis of Hansen’s disease, providing a specific target to identify *M. leprae*.^10^ Currently, the PGL-1-based ELISA test still contributes to determining whether a patient is free from leprosy bacilli.^41^ In addition to its importance in the process of infection, the PGL-1 saccharide fraction is species-specific for *M. leprae,* constituting a highly immunogenic molecule that is quickly recognised by the immune system.^42^ Thus, the conjugate composition of PGL-1 has been associated with its neural tropism from *M. leprae*.^8^

The domains present in the LAMA2 protein, including a set of EGF-laminin domains, are essential for signal transduction and protein dimerization.^13^ The LN domain is involved in basement membrane assembly, and its role might be a cooperative process in which laminins polymerise through their N-terminal domain (VI) and anchor to the cell surface. Finally, LamG was associated with cellular adhesion to laminins and was mediated by a repetitive region of five laminin G-like (LG) domains.^43^ ^,^ ^44^ Consistently, our peptide was designed on the basis of the C-terminal of the LAMA2 chain, which contains five LamG domains, in which PGL-1 seems to contact LAMA2 during the mycobacterial invasion of Schwann cells.^8^ ^,^ ^45^

Our peptide contained a portion of the LamG domain, and further analysis showed that it is compatible with the steroid-binding site of related proteins containing laminin G-like domains, including those with sexual hormone-binding sites that resemble lipid-related ligands.^46^ In this study, we proposed that this site might anchor PGL-1 to the cell surface through LAMA2, representing the initial bacilli-specific interaction. Consistently, a study has shown that *M. leprae* is strongly bound to the LAMA2 C-terminal but not to the N-terminal region in the proximal G1-G3 subdomains.^12^ ^,^ ^47^

In this paper, we attempted to identify the region from human LAMA2 that was necessary for triggering the attachment of *M. leprae* to Schwann cells. Our computational peptide design supported the 60-residue simulation that might be sufficient to capture peptide folding.^48^ The peptide encompassing residues 2161-2220 of LAMA2 might be able to bind flexibly to PGL-1, allowing bacterial attachment and subsequent pathogenesis. In our MD simulations, the peptide conformation was more stable in the presence of PGL-1 than in the absence, which hypothetically seems to be an early step in bacilli membrane attachment. Additionally, our simulations suggest that PGL-1 prefers to interact with one of the two faces of the peptide, i.e., the face called the front face in this study. For example, PGL-1 might be positioned by a network of van der Waals and hydrophobic interactions in the loop region and residues located in three of the four main beta-sheets. The docking and simulation results also supported that the binding of PGL-1 to the LAMA2-derived peptide is flexible on its N-terminal region (ΔG -5.1 Kcal/mol) and, thereby, might increase the affinity between the ligand and the peptide or even homodimers. Our peptide-aided design in the selection of peptide candidates promotes hydrophobic interactions that might define the PGL-1 binding site and proper adjustment to the basal laminin of Schwann cells. Similarly, hydrophobic interactions contribute strongly to steroid pocket binding and fine-tuned interactions with hydrophobic ligands in proteins containing laminin-like domains.^46^ ^,^ ^49^ Our molecular dynamic results suggested that a flexible loop region assisted as the gate for PGL-1 (through interactions with residues, such as Tyr7 and Val11), as reported similarly in sex steroid hormones, resembling the capability to bind lipidic ligands with their loop segment for ligand-specific rearrangement.^49^

Protein interactions with cellular membranes have been thoroughly studied as computational models in antimicrobial peptides.^50^ ^,^ ^51^ However, we performed peptide-glycolipid molecular modeling predictions as a novel approach for antimicrobial peptide design against a key conjugate molecule from leprosy bacilli. Our calculations included the possibility of peptide homodimerization and homodimer interaction with PGL-1. Although the predicted affinity for the homodimer was lower (4.8 kcal/mol) than that for the peptide alone (5.1 kcal/mol), it is necessary to mention that this bond is flexible; that is, this affinity can vary over time and, as expected, depends on the conformational changes that occur in the receptor. Interaction percentages less than 40% and a variety of structural conformations were reported for the organic compounds that interacted with highly flexible proteins.^52^ Given that we found higher percentages of interaction and a high conformational stability, our results are promising.

The LAMA2-based peptide might interact with membranes and has a chance to be an antimicrobial peptide in which aligning is performed to find the most similar peptides in the APD database. This peptide showed a 32% similarity with halocin-like peptides (halocin S8 and halocin R1), which have activity against Gram-positive and Gram-negative bacteria and a similar hydrophobic ratio.^53^ The results showed that peptides with a low similarity (below 30%) presented a shared hydrophobicity percentage with PGL-1 based on the sequence comparison, which was performed to find antimicrobial peptides that most resembled our input peptide sequence in the APD. Consistently, previous approaches with host-based peptides were tested successfully to control mycobacterial growth, and hydrophobicity was determined to be a key parameter for enhancing mycobactericidal activity and selectivity.^54^ ^,^ ^55^ ^,^ ^56^ ^,^ ^57^

Our findings might contribute to deciphering the first step of how *M. leprae* establishes initial host contact for manipulating signaling pathways leading to axonal damage and hindering myelin maintenance in the basal lamina.^58^ Similar mechanisms have been reported involving other host receptors that contain laminin-like domains for the entry of viral pathogens.^59^ ^,^ ^60^ For example, the virus that causes Lassa fever targets Schwann cells, selectively interfering with the myelination process through its viral receptor dystroglycan, leading to neurological disorders.^61^

Our study might provide insights into how mycobacterial glycolipids interact with host laminin and suggests a new strategy for exploring the development of new diagnostic or therapeutic options based on the druggable proteome from the leprosy bacillus.^62^ ^,^ ^63^ ^,^ ^64^

*In conclusion* - We identified N-glycosylation sites, disulfide bridges, and domains along human LAMA2. We dissected the LAMA2 sequence into 52 peptides, including the potential PGL-1 binding site from *M. leprae*. Our peptide targeting PGL-1 is located between residues 2161-2220, and its structure exhibits a combination of β-sheets and random coiled region that might flexibly bind PGL-1. Thus, our approach with a specific peptide could block the interaction of *M. leprae* with the host cell, thereby preventing long therapeutic regimens, disease chronicity, and possibly nerve damage in Hansen’s disease patients.
